# Human brain harbors single nucleotide somatic variations in functionally relevant genes possibly mediated by oxidative stress

**DOI:** 10.12688/f1000research.9495.3

**Published:** 2017-01-12

**Authors:** Anchal Sharma, Asgar Hussain Ansari, Renu Kumari, Rajesh Pandey, Rakhshinda Rehman, Bharati Mehani, Binuja Varma, Bapu K. Desiraju, Ulaganathan Mabalirajan, Anurag Agrawal, Arijit Mukhopadhyay

**Affiliations:** 1 Genomics & Molceular Medicine Unit, CSIR-Institute of Genomics & Integrative Biology, Delhi, 110020, India; 2 Academy of Scientific and Innovative Research, CSIR-Institute of Genomics & Integrative Biology (AcSIR-IGIB), Delhi, 110020, India; 3 CSIR Ayurgenomics Unit- TRISUTRA, CSIR-Institute of Genomics & Integrative Biology, Delhi, 110020, India; 4 Molecular Immunogenetics Unit, CSIR-Institute of Genomics & Integrative Biology, Delhi, 110020, India; 5 School of Environment and Life Sciences, University of Salford, Manchester, UK

**Keywords:** somatic variations, exome sequencing, oxidative stress, 8-OHdG, brain, SNVs, axon guidance, G:C>T:A transversions.

## Abstract

Somatic variation in DNA can cause cells to deviate from the preordained genomic path in both disease and healthy conditions. Here, using exome sequencing of paired tissue samples, we show that the normal human brain harbors somatic single base variations measuring up to 0.48% of the total variations. Interestingly, about 64% of these somatic variations in the brain are expected to lead to non-synonymous changes, and as much as 87% of these represent G:C>T:A transversion events. Further, the transversion events in the brain were mostly found in the frontal cortex, whereas the corpus callosum from the same individuals harbors the reference genotype. We found a significantly higher amount of 8-OHdG (oxidative stress marker) in the frontal cortex compared to the corpus callosum of the same subjects (p<0.01), correlating with the higher G:C>T:A transversions in the cortex. We found significant enrichment for axon guidance and related pathways for genes harbouring somatic variations. This could represent either a directed selection of genetic variations in these pathways or increased susceptibility of some loci towards oxidative stress. This study highlights that oxidative stress possibly influence single nucleotide somatic variations in normal human brain.

## Introduction

Somatic variations are an inevitable consequence of continuous cell divisions in multicellular complex organisms and can lead to genomic heterogeneity. Somatic variations can arise due to replication error with or without external environmental factors like mutagens, exposure to UV rays etc – and accumulate over time as the organism ages. Depending on how early a somatic variation occurs in a particular cell lineage and the rate of division for that cell, somatic variations may clonally expand and cross the threshold of detection by genome sequencing technology. As reviewed by De, somatic variations can range from single nucleotides to whole chromosomes and can be found in both ‘healthy’ and ‘diseased’ tissues – cancer being a unanimously accepted example ^1^. The contribution of somatic variations is widely reported in cases where the DNA from affected tissue was found to harbor causal mutations whereas they were absent in the DNA from peripheral blood ^2– 4^. Mutation reversal due to somatic variation has also been reported in Mendelian diseases, indicating the stochastic nature of these variations ^5^. The rates of somatic variations have been a matter of debate, ranging between 10 ^-4^ to 10 ^-8^ per base-pair, per generation, depending on whether these estimates were genome-wide (lower estimates) or locus-specific (higher estimates) ^6, 7^. In addition, it has been speculated that rates of somatic variations might differ for different tissue-types and different developmental times but this remains to be clarified ^1^.

Somatic variations acquired and accumulated during the course of development have been modelled, predicting a higher risk of cancer and neurodegenerative diseases. The multitude of possible outcomes would increase with increased complexity of the tissue type ^8– 10^. Thus, somatic variations could be of great importance for an organ like mammalian brain, which has complex structural and functional organization, high plasticity, and limited regenerative capabilities. The extreme interconnectivity of cortical neurons permits a disproportionately large impact of small changes while retaining robust adaptive mechanisms. Recently, there have been attempts to explore the extent of somatic variations in diverse healthy human tissues at the level of microarray or deep sequencing based copy number changes ^11, 12^. Interestingly, normal human brain, especially in the neuron rich regions, has been shown to harbor a wide variety of somatic variations – ranging from whole chromosomes ^13, 14^, large-scale retro transpositions ^15, 16^, and copy number variations at the single neuron level ^17^. Recent reports have also started revealing the importance of such variations in neurological disorders ^10^. But there have been very few systematic studies so far exploring the nature, extent and impact of somatic variations at the single nucleotide resolution in neuron-rich parts of the healthy human brain ^18, 19^.

In this study, we have analyzed single nucleotide level somatic variations between frontal cortex (rich in neurons) and corpus callosum (lack neurons) from healthy individuals and correlated the findings with markers of oxidative stress.

## Methods

### Sample collection and DNA isolation

Paired tissues were taken from a total of nine individuals, with age ranging from 23 years to 45 years ( Supplementary table S1). For four individuals (post-mortem, road accident victims), tissue sections from two different parts of the brain viz. frontal cortex (FC) and corpus callosum (CC), were procured from NIMHANS Brain Bank, Bangalore, India. For the other five individuals, peripheral blood and saliva was taken from healthy volunteers representing circulatory cell types with high turnover and therefore high likelihood of spontaneous somatic variations. The project was approved by institutional human ethics committee of CSIR-Institute of Genomics and Integrative Biology and adhered to the international ethical guidelines (Declaration of Helsinki). For brain tissues, DNA was isolated using Omniprep Genomic DNA isolation kit (G-Biosciences, USA) and DNA from two other cell types viz. leukocytes (from blood) and epithelial cells (saliva) were isolated using Qiagen DNA kit (Qiagen, USA) and Oragene Saliva kit (DNA Genotek, Canada), respectively using manufacturer recommended protocols.

### Exome sequencing of different tissues and data analysis

Exome capture for the isolated DNA was done using Illumina TruSeq Exome capture kit (62 Mb) and further exome libraries were sequenced (100 bp paired end) using Illumina Hiseq 2000 (Illumina, USA). Exome sequencing was done using manufacturer recommended protocols. A total of ~1.5 billion reads were generated for all the 18 samples. Supplementary figure S1 represents the overall pipeline followed for the analysis of the data and further text explains each step in detail. Raw data was checked for per base quality score and reads having 80% bases with phred quality score 30 (Q30) and greater were carried forward for downstream analysis and rest were discarded. Also, last few low quality bases were trimmed from all the reads (4–10 bases, depending upon the sample quality). This was done using Fastx (version 0.0.13) and FastQC (version 0.10.0). About 9–14% of data was removed from each sample. After checking quality of the data in various aspects, reads (Read 1 and Read 2 for each sample) were aligned to the reference genome (hg19) using BWA (version 0.6.1) ^20^ allowing for maximum 2 mismatches. More than 98% percent of the data was aligned to reference for each sample. Data was also checked for PCR duplicates and the same were removed. Only reads with mapping quality (MQ) more than 40 were taken forward for further analysis. The sequence depth for the samples ranged between 91×–120× (average 100×) for the FC-CC samples and 25×–86× (average 51×) for the blood saliva samples ( Supplementary figure S2). Data has been deposited in the NCBI Sequence Read Archive and can be found at accession number SRP045655.

### Somatic variation analysis

Varscan2 ^21^ (version 2.3.5) somatic module along with Samtools (version 0.1.18) ^22^ was used to call somatic variations from all the paired samples (CC vs. FC and blood vs. saliva). Firstly .bam files were processed using Samtools for making .mpileup files which were further processed through Varscan2 to call somatic variations using somatic SNP module. Following parameters were considered while calling variations:

- Minimum coverage to call somatic variation at a locus was kept at 8 reads
- Minimum variant frequency to call a heterozygote was kept at 0.1 of total reads for that position.
- Minimum variant frequency to call a homozygote was kept at 0.90 of total reads for that position.
- Variants with more than 90% strand bias were removed
- Minimum base quality score was kept at 20
- Minimum mapping quality of a read was kept at 40

After calling variations, the data was checked for read bias. In this filter we excluded variations for which all the reads with the variant allele were read only from one direction (either F1R2 or F2R1) using in house developed perl scripts. Variations with more than 90% reads from one strand were removed. Annotation of all the variations called was done using Annovar (version 2012-03-08) ^23^ and VcfCodingSnps (v1.5). Sites falling in regions with 87% and above identity with another genomic region were also removed from the data. Sites were termed as somatic sites if they had different genotype in two tissues and somatic p-value was less than 0.05. Overall out of a total 371 somatic sites (with p<0.05) in brain 93.8% had at least 4 reads for the variant allele. All the sites confidently called as same genotype in both tissue types (p<0.05) were termed as germline variations. Further, somatic variations with supporting reads for the variant allele >10% of total reads were considered as heterozygotes and those with <5% reads supporting variant allele were called homozygotes. All the somatic sites with their details are provided as CSV files under ‘Data availability’. Percentage of somatic variations across different sample-pairs was calculated by the following formula: (number of somatic variations/number of total variations) × 100. Number of somatic sites were defined as sites with different genotype between two tissues of the same individual. and Nnumber of total variations was refers to, all sites that had varying genotype from the reference genome (hg19) in two tissues of the same individual. We also used MuTect ^24^ (version 1.4.4) variant caller to call somatic variations from exome sequencing data for the brain samples. Details of the concordance between the two platforms is described in Supplementary table S2. It was found that up to 78.8% of the somatic sites called by Varscan2 were also called as somatic sites by MuTect. Concordant sites between the two software also showed an enrichment of G:C>T:A transversions with FC harboring heterozygotes. Pathway analysis of somatic variations was done using Gene Set Enrichment Analysis ^25^ (release 2.2.0). The total 359 genes harbouring somatic variations from all brain samples (from varscan2 dataset) were included for pathway analysis. Pathway analysis for the concordant sites also showed similar results.

### Detection of neurons in brain samples

Western blotting for NeuN: Total tissue lysates from FC and CC were made using radio-immuno precipitation assay (RIPA) buffer from 5 samples (3 samples viz. Brain_152, Brain_156 and Brain_202 that were sequenced and extra 2 samples viz. Brain_174 and Brain_119 that were not sequenced). Two extra samples were considered to emphasize that this contrast in NeuN between FC and CC tissue is true across all samples and is not specific to only the ones that were sequenced. Protein estimation was done using bicinchoninic acid assay method (BCA method) and 30 micro gm (ug) of protein was used for western blotting for NeuN, which was selected as marker for detecting neuronal cell bodies in FC and CC. Anti-NeuN monoclonal antibody (ab104224, Abcam), raised in mouse was used at a dilution of 1:1000. This antibody gives 2–3 bands between 46–48 kDa as per manufacturer data-sheet, which we also observed. GAPDH (2118L- Cell Signalling, 1:2000) was used as loading control. For this experiment a 5% stacking gel and a 12% resolving gel were used (both with 30% acrylamide, all reagent were from Sigma-Aldrich, USA). Images were analyzed by Image J 1.48 version.

Immuno-Fluorescence for NeuN: The same antibody was used to do immuno-fluorescence in both the tissues. Immunofluorescence for NeuN was performed on 8 µm sections of FC or CC. Briefly, tissue sections were fixed in chilled acetone, permeabilized with 0.1% Triton X-100, blocked in 1.5% FBS, incubated with Anti-NeuN monoclonal antibody (1:500, 16–18hrs at 4°C) and anti mouse Alexa 488-conjugated secondary antibody (1:200, 1hr at RT), mounted with DAPI containing solution before taking images using a confocal microscope (at 63×) [Zeiss-510 Meta, Carl Zeiss, Zen-2009 software (December 2010 release)].

### ELISA for 8-OHdG

8-OHdG, oxidative DNA damage marker, was measured in lysates of FC and CC by competitive ELISA (Cayman, USA). Briefly, 3 µg lysates were incubated with conjugate and antibody for 18 hrs and developed using Ellman’s reagent and absorbance readings was taken at 405 nm and results were expressed in pg/3 µg for each lysate.

### Amplicon sequencing for validation of somatic sites

We randomly selected 20 sites from Brain_156 (15% of the total sites) due to availability of sufficient DNA and this pair having the highest number of somatic sites, for validation using Illumina MiSeq Low Sample protocol (LS). Amplicons ranging from 200 bp to 350 bp for 14 sites were generated using PCR. The PCRs were done for 35 cyles with annealing temperatures at either 62 or 58 degrees C (primer details are provided under ‘Data availability’). Further all the amplicons from each tissue-type were pooled together in equimolar ratios, resulting in 2 freshly prepared libraries. These pooled amplicons were then processed using LS protocol of MiSeq sample preparation kit. About 12 million total reads were obtained from the two libraries. Quality check of trimming 3’ ends of the reads and filtering reads with less than 80% bases with Q30 phred quality score was applied on the raw data. Further, frequency of the variant allele (as observed in HiSeq data) was checked in both the tissues (FC and CC) in MiSeq data. A site was considered validated if the variant allele was supported by minimum of 100 reads along with at least two fold differences in the variant allele frequencies between the two tissues. On an average read depth of 5000× was obtained across all sites captured.

## Results

Raw data for ‘human brain harbors single nucleotide somatic variations in functionally relevant genes possibly mediated by oxidative stress’ README.txt contains a description of the files. Click here for additional data file. Copyright: © 2017 Sharma A et
al. 2017 Data associated with the article are available under the terms of
the Creative Commons Zero "No rights reserved" data waiver (CC0 1.0
Public domain dedication).

We analyzed about 1.5 billion reads from whole exome sequencing of 18 samples each having ~60 Mb coverage and identified 2,49,607 single nucleotide variations (SNVs), with an average of ~27,700 sites per sample ( Supplementary figure S3). For technical confidence of the genotype calls all the samples were genotyped on Illumina Infinium 660W-Quad microarrays and we observed 99.8%–99.9% genotype concordance between the NGS and the microarray data ( Supplementary table S3).

### Human brain harbors higher somatic variations compared to circulatory tissue with a bias for non-synonymous changes

**Figure 1.**
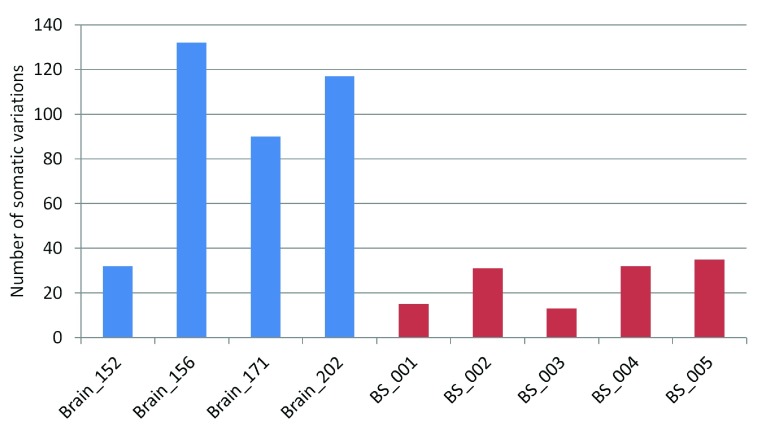
Number of somatic variations in brain and blood saliva samples. Blue bars represent brain samples and red bars represent blood-saliva samples.

The number of high-confidence somatic variation calls seen for each pair ranged between 32–132 for the FC-CC pairs while it varied between 13–35 for the blood-saliva pairs ( Figure 1). The percentage of somatic variations ranged between 0.1%–0.48% for the brain sample-pairs (FC-CC) and between 0.03%–0.17% for the blood-saliva pairs. The number of germline variations across all samples was comparable ( Supplementary figure S3). The observed somatic variations do not show any bias for the position of the variant loci. The distribution was 37–65% in CDS, up to 57% in 3’ and 5’ UTRs and a minor proportion from other regions such as non-coding RNAs, splice sites etc. A comparison of these distributions between germline and somatic variations is represented in Supplementary figure S4.

**Figure 2.**
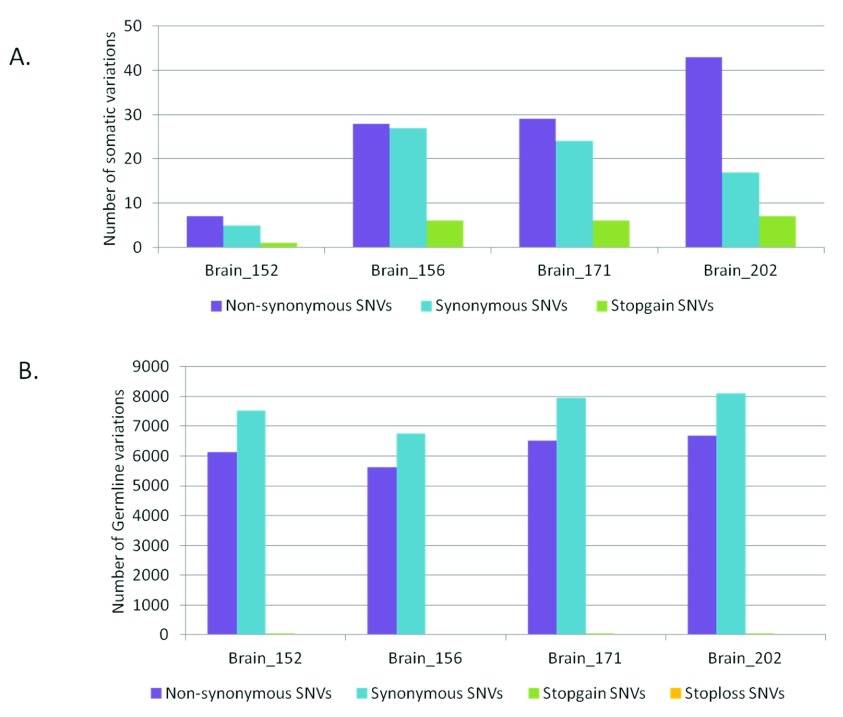
Distribution of synonymous and non-synonymous variations in brain samples **A**) for somatic variations and **B**) for germline variations.

As much as 64% of the somatic variations in the brain samples would lead to non-synonymous changes at the protein level ( Figure 2A). Whereas, for the germline variations the trend was towards higher synonymous variations as expected ( Figure 2B). For the blood-saliva pairs such a consistent trend towards non-synonymous variations for the somatic sites was not observed ( Supplementary figure S5). We analyzed the possible effect of the somatic sites at the amino-acid level for all samples and did not find a consistent bias for any particular amino acid change ( Supplementary figure S6).

### Enrichment of G:C>T:A somatic transversions in the brain

**Figure 3.**
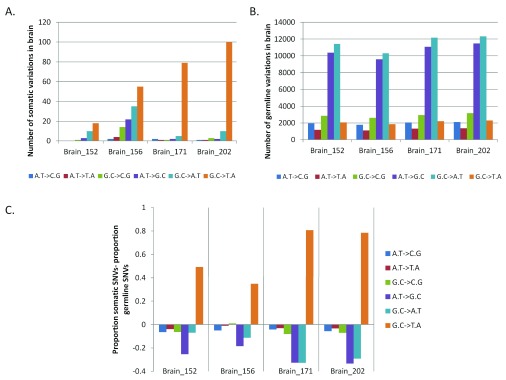
Enrichment of G:C>T:A somatic transversion events in brain. For each pair of FC-CC samples the absolute numbers of different types of changes are plotted for somatic ( **A**) and germline variations ( **B**). ( **C**) The difference in proportion between somatic SNVs to germline SNVs is plotted for the brain samples. The positive values on the vertical axis denote enrichment of the type of variation in the somatic subset while a negative value indicates enrichment of the type of variation in the germline subset. As evident in the figure only the G:C>T:A transversions show enrichment for the somatic variations in brain.

Having observed somatic variation in the brain with a trend towards higher non-synonymous changes, we analyzed the level of transversion over transition amongst the somatic sites. Up to 87% of the total somatic variations found in FC-CC pairs were G:C>T:A transversions ( Figure 3A)! The germline variations (for all samples) matched the expected and reported distribution where A:T>G:C (36%) and G:C>A:T (38%) transitions were the most common types of changes ( Figure 3B). In Figure 3C we have represented the enrichment of G:C>T:A transversion events as a proportion of somatic variations, with respect to the germline variations. The blood-saliva pairs did not show such consistent positive enrichment for the same class of transversion events ( Supplementary figure S7).

**Figure 4.**
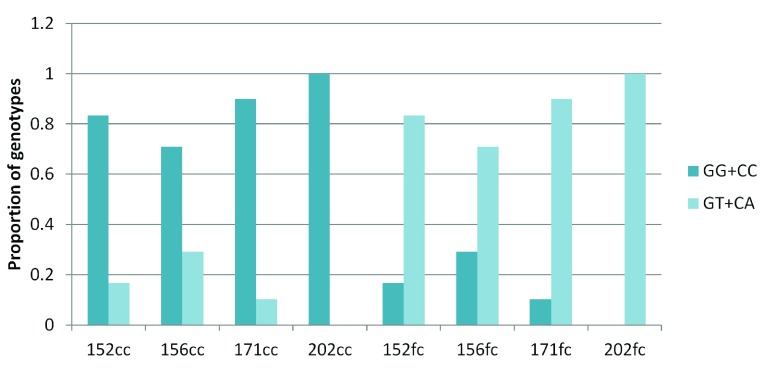
Enrichment of GT and CA heterozygotes in the frontal cortex: The figure shows that across the four pairs, for the somatic G:C>T:A transversion sites majority of the heterozygotes (variant alleles) were present in the frontal cortex while the corresponding corpus callosum DNA had the homozygote (reference) genotype.

Surprisingly, for the G:C>T:A transversion sites, 70–100% of GT and CA heterozygotes were present in the frontal cortex and the corresponding homozygotes were observed in the corpus callosum ( Figure 4). For all other sub-classes of somatic variations in brain and blood-saliva pairs the distribution of reference homozygotes versus heterozygotes did not show such a consistent and overriding bias ( Supplementary figure S8).

**Figure 5.**
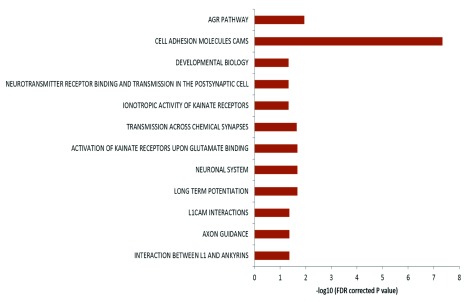
Pathway enrichment analysis of genes harboring somatic variations: Enrichment of genes harboring somatic variations in brain for pathways related to neuronal function. In the figure the horizontal axis shows the negative logarithm of FDR corrected p-value. Different biological processes are indicated on the left.

The genes harboring the somatic variations in all the brain samples were analyzed for canonical pathways. It was observed that the genes were enriched for axon guidance pathways and neuronal processes related pathways ( Figure 5) with p-values ranging from 0.04 to 4.9×10 ^-8^ (FDR corrected). When analyzed by individual samples, three out of four samples also showed similar enrichment – but for Brain_152 although the relevant genes were found in the dataset but due to small number of variations, enrichment could not be established. It has been shown that DSBs in neuronal cells tend to occur in long genes involved in neuronal functions ^26^. We also find a similar trend in our data: 46% of the pathway genes harboring somatic variations were long (>100kb) compared to total genes (18% are >100kb, p<0.002).

### Increased accumulation of oxidative stress mediated modified guanine in frontal cortex

**Figure 6.**
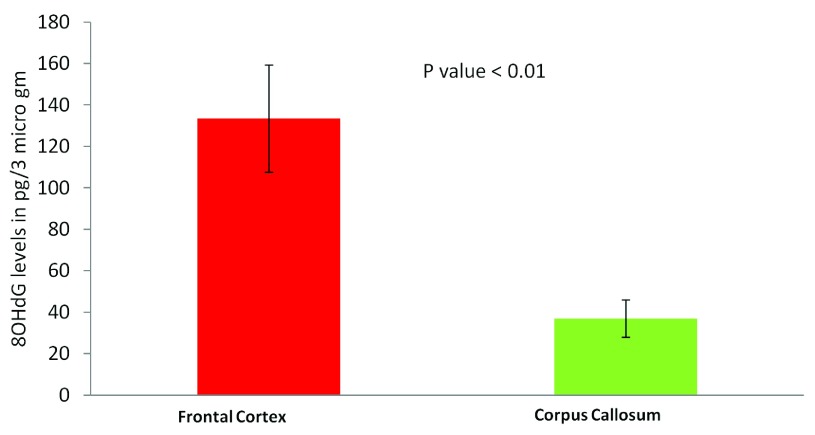
Estimation of 8-OHdG levels in different brain tissues using ELISA: Frontal cortex samples show significantly higher 8-OHdG accumulation. The figure represents concentration of 8-OHdG in the frontal cortex and corpus callosum samples. For each tissue type five independent samples were analyzed by ELISA. The error bars represent standard deviation (SD for FC, 57.5 and SD for CC, 24.4). The p value is calculated using paired T-test.

**Figure 7.**
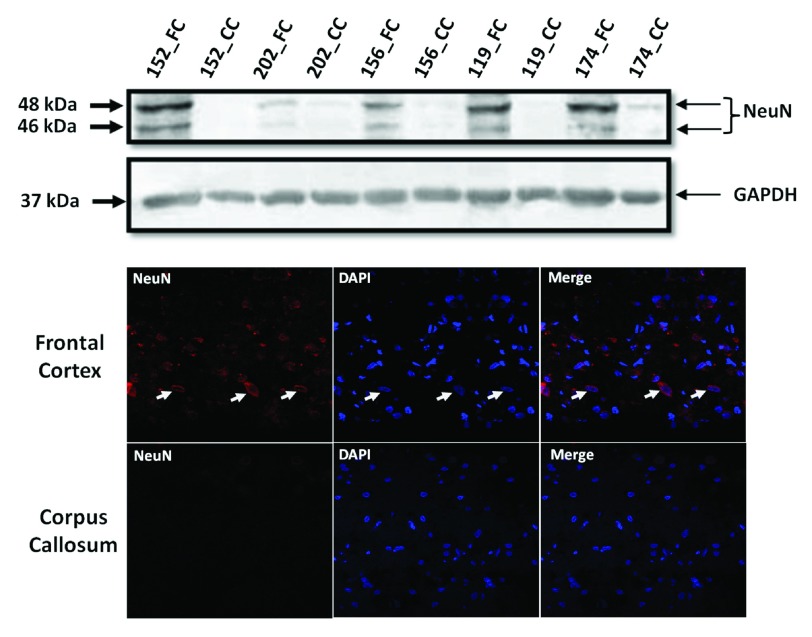
Frontal cortex is enriched for neuronal cells: ( **A**): Western blot for NeuN in five pairs of FC-CC samples (for three of them sequence data is presented) shows distinct abundance of neurons in the FC in contrast to CC. GAPDH expression is used as a control. ( **B**): The same antibody is used in immuno-fluorescence to show the neuronal abundance in FC. Note the near absence of signal for NeuN in the corpus callosum panel.

The most common cause of G:C>T:A transversions is mis-pairing of G to A (instead of G to C) due to modification of deoxy guanosine (dG) to 8-hydroxy-2’-deoxy-Guanosine (8-OHdG) mediated by oxidative/metabolic stress ^27– 29^. We found significantly higher levels of 8-OHdG in the frontal cortex samples, when compared to the corpus callosum of the same individuals ( Figure 6), which corresponded with the abundance of neuronal cells in the frontal cortex ( Figure 7). Thus an increased accumulation of 8-OHdG in the frontal cortex (compared to the corpus callosum of the same individual) DNA might result in localized DNA variations with a bias towards G:C>T:A transversions. Using immunoblotting and immuno fluorescence staining on tissues against the neuron specific marker NeuN we confirmed that the majority of the cells in the FC samples were neurons whereas, CC samples were almost devoid of neurons ( Figure 7) – implicating a direct correlation of abundance of neuronal cells with accumulation of 8-OHdG leading to G to T somatic transversions in the FC samples. Further effect of postmortem could be ruled out as one of the contributing factors, as time of collection of tissues, storage conditions and DNA isolation protocol were exactly the same for both the tissues.

### Validation of somatic variations using targeted amplicon resequencing

Amplicon sequencing using MiSeq (Illumina) was performed for validation of the somatic variations. Out of the total somatic sites across all brain samples 20 sites from sample number Brain_156 were chosen for validation. We have randomly selected 20 sites from 156 (15% of the total sites) due to availability of DNA and this pair having the highest number of somatic sites. Data for 14 sites was obtained and 10 out of these 14 sites showed difference in variant allele frequencies between the two tissues (CC vs FC) in accordance with the HiSeq data (71% validation rate) ( Supplementary Table S4). Rows marked in red shade are the ones that got validated between two platforms. It should be noted that although the allele frequencies of the variations in the validation set were not exactly the same as in the HiSeq data but the trend (same tissue harboring the higher amount of variant allele) was same. In general, low variant allele frequencies were obtained in MiSeq data as compared to HiSeq data. This could be attributed to very high read depth per site in MiSeq data. A trend of varying allele frequencies with read depth was observed, as in, lower the read depth higher the allele frequencies (and vice versa), thereby explaining the high frequencies in HiSeq data (with average read depth of 100×) as compared to MiSeq data (with average read depth of 5000×).

## Discussion

Here, we report somatic variations in normal human brain at single base resolution in whole exomes. Earlier studies have reported somatic genomic rearrangements such as, aneuploidy, insertions/deletions, CNVs etc in the neurons as part of the normal brain development and neurogenesis ^9, 13– 17, 30– 32^. In our data the percentage of somatic variants in brain is 0.1%–0.48%, which was significantly enriched for genes in axon guidance (details below). It has been reported that somatic events present in low abundance of the cells can bring about striking phenotypic consequences in the brain ^10, 33^. We observed an enrichment of somatic non-synonymous variations, which was unique and was not found in variants common to both tissues (germline variations, Figure 2) – implying a functional neutrality or advantage of such variants. In absence of additional tissues and parental information for the individuals, we cannot definitively distinguish between inherited and acquired genotypes.

 We have estimated the error rates to rule out that most of our variations can arise due to errors in sequencing experiments. Towards this we have performed genome-wide genotyping of the same samples and genotype discordance (error rates) between the microarray and the sequencing data varied from 0.002 to 0.0008 ( Supplementary table S3). Our observed somatic site frequencies were higher than that. In addition, to further strengthen the criteria, we have chosen to only accept those sites as variants, where the variant allele was represented by at least 10% of the total reads for that position. We also performed targeted amplicon sequencing to validate a subset of the somatic sites.

We observed a higher proportion of somatic variations between the FC-CC samples compared with the proportion found between blood-saliva samples. A recent report studied somatic sites between brain and the blood of the same individual and found higher somatic sites in the blood ^34^. This apparent contrast in the two findings is perhaps due to the fact that in our study we did not compare between blood and brain of the same individuals. In addition, a lower proportion of somatic variations between DNA from blood and saliva can be due to, inter-mixing of the two cell types and/or faster regeneration and circulatory nature of these cells resulting in dilution of clonal populations of cells harboring the somatic sites.

Our data shows all possible types of nucleotide changes amongst the somatic SNVs, as would be expected for a random event, but with an unexpected bias (up to 87%) for G:C>T:A transversions ( Figure 3A). Moreover, more than 70% of these transversion events were found in the frontal cortex while DNA from the corpus callosum harbored the homozygotes for the reference alleles ( Figure 4). Recently, two studies ^18, 19^ have looked into somatic SNVs at the single neuron level. Although, neither of these studies report enrichment of G:C>T:A transversions, but these transversions are the second most abundant class of somatic variations as reported by Hazen *et al.* ^19^ (16% of total somatic variations) in their dataset. Moreover, the choice of tissue in these two studies (single neurons) and ours (frontal cortex v/s corpus callosum) is different, which might be the reason for differences between the classes of somatic variations observed.

It is well known that G:C>T:A transversions are mediated primarily by oxidative stress which modifies deoxy-guanine (dG) to 8-hydroxy-2’-deoxy-Guanosine (8-OHdG) ^27^. Towards this when probed for oxidative stress levels in two tissues, we also found significantly higher levels of 8-OHdG in the frontal cortex compared to the same individual’s corpus callosum correlating the enriched transversion events with increased oxidative stress ( Figure 6). A recent study reported G:C>T:A transversions arising in sequencing data as an artefact due to DNA shearing stress ^35^. We have tested for this bias in both the germline and somatic datasets. A major fraction of the somatic sites initially called by our pipeline was observed to have this bias and were removed in the modified analysis workflow. However, it is still possible that in the remaining data-set presented here, some of the G:C>T:A transversions are actually artefacts. Interestingly, even in such a possible scenario, these artefacts are not randomly observed in all tissue types analysed unlike the earlier report ^35^. Instead, we observed that almost exclusively the GT and CA heterozygotes were in the FC samples. Further, the observation of higher 8-OHdG in FC was independent of the shearing stress as the experiments were performed on lysates isolated from fresh sections of the same tissue samples. Whether the specific cell types in FC make them more prone to either *in-vivo* (biological) or *in-vitro* (artefactual) stress mediated variations needs to be explored further. 

It is known that having an Adenine (A) 3’ to the oxidized G significantly reduces the efficiency of the repair process and thereby enhancing the possibility of a G>T transversion ^36^. We also find a bias for 3’ Adenine for the somatic G:C>T:A sites in our data ( Supplementary figure S9). As reported in the above-mentioned study, the artefactual C>A transversions have an enrichment of C CG motif, which perhaps makes the base (underlined) more susceptible for oxidation ^35^. We did not observe any enrichment for this motif for the somatic sites found in our study.

Our data indicates that normal brain accumulates single nucleotide somatic variations with age during the lifetime of an individual. This might happen due to various mechanisms, high oxidative stress generated during normal physiological brain activity, being one of them. Physiological levels of oxidative free radicals are essential in various key cellular processes such as cellular differentiation, proliferation and survival ^37^ though pathological level is detrimental for cellular health. From our observed results, it seems that most of the variations appeared around the time of birth when neurons are rapidly dividing. It is known in the literature that the process of neurogenesis spans from E13 to E108 ^38^ and the number of neurons in an infant brain is in the order of 10 ^10^ – 10 ^11^. With our analysis threshold of at least 10% abundance of the variant allele, the variation should be present in 10 ^6^–10 ^7^ cells – which is in order with the developmental time-frame. However, studies with more tissues (from brain and outside brain) from the same individuals would be needed to rule out other possible reasons. Oxidative stress is also induced during normal neurogenesis in adults ^39^ and oxidative stress susceptible genetic alleles in drosophila are connected to axon guidance ^40^. A recent study showed that physiological levels of H _2_O _2 _are essential for neurogenesis. Their data revealed that exposure to H _2_O _2_ mediated oxidative stress promoted neurogenesis of neural progenitor cells (NPC) in rat ^41^. In this context, interestingly, the somatic transversion events we found in brain samples were enriched for genes involved in processes like axonal guidance, neurogenesis etc. ( Figure 5). These indicate that the accumulation of somatic variations could be the possible molecular explanation for physiological oxidative stress mediated enhancement of neuronal differentiation from NPCs. Other linked processes like interaction between L1 and ankyrins, NCAM signaling, long term potentiation etc. were also found to be significantly enriched. These evidences indicate that the acquired somatic variations might provide required functional diversity in the growing neurons during development as well as during adult neurogenesis.

## Conclusions

Our study shows presence of somatic SNVs in functionally relevant genes in different parts of the brain possibly influenced by oxidative stress along with other known contributing factors. Recent reviews suggested that local somatic events could strike a balance between the plasticity and robustness of the genome indicating a continuum of normal-through-disease scenario ^1^. A study showed that oxidative stress mediated double-strand breaks (DSBs) in DNA of neuronal cells and its delayed repair was a feature of normal mice brain related with its learning ability ^42^. On similar lines our study also indicates that the acquired somatic variations might provide required functional diversity during development as well as during adult neurogenesis.

## Data availability

The data referenced by this article are under copyright with the following copyright statement: Copyright: © 2017 Sharma A et al.

Data associated with the article are available under the terms of the Creative Commons Zero "No rights reserved" data waiver (CC0 1.0 Public domain dedication). 

Data has been deposited in the NCBI Sequence Read Archive under accession number SRP045655.

F1000Research: Dataset 1. Raw data for ‘human brain harbors single nucleotide somatic variations in functionally relevant genes possibly mediated by oxidative stress’., 10.5256/f1000research.9495.d149136 ^43^
