# Supplementary material for: Human brain harbors single nucleotide somatic variations in functionally relevant genes possibly mediated by oxidative stress
Source: F1000Res. 2017 Jan 12;5:2520. Originally published 2016 Oct 14. [Version 3] doi: 10.12688/f1000research.9495.3 (PMC5265704; doi:10.12688/f1000research.9495.3)
Supplement: Supplementary file 2 [file f1000research-5-11473-s0001.tgz › 995053d4-b9c3-4ed5-bd6f-e2799d80c632.docx]

**Supplementary Table S1: Details of the samples included in the study**

**
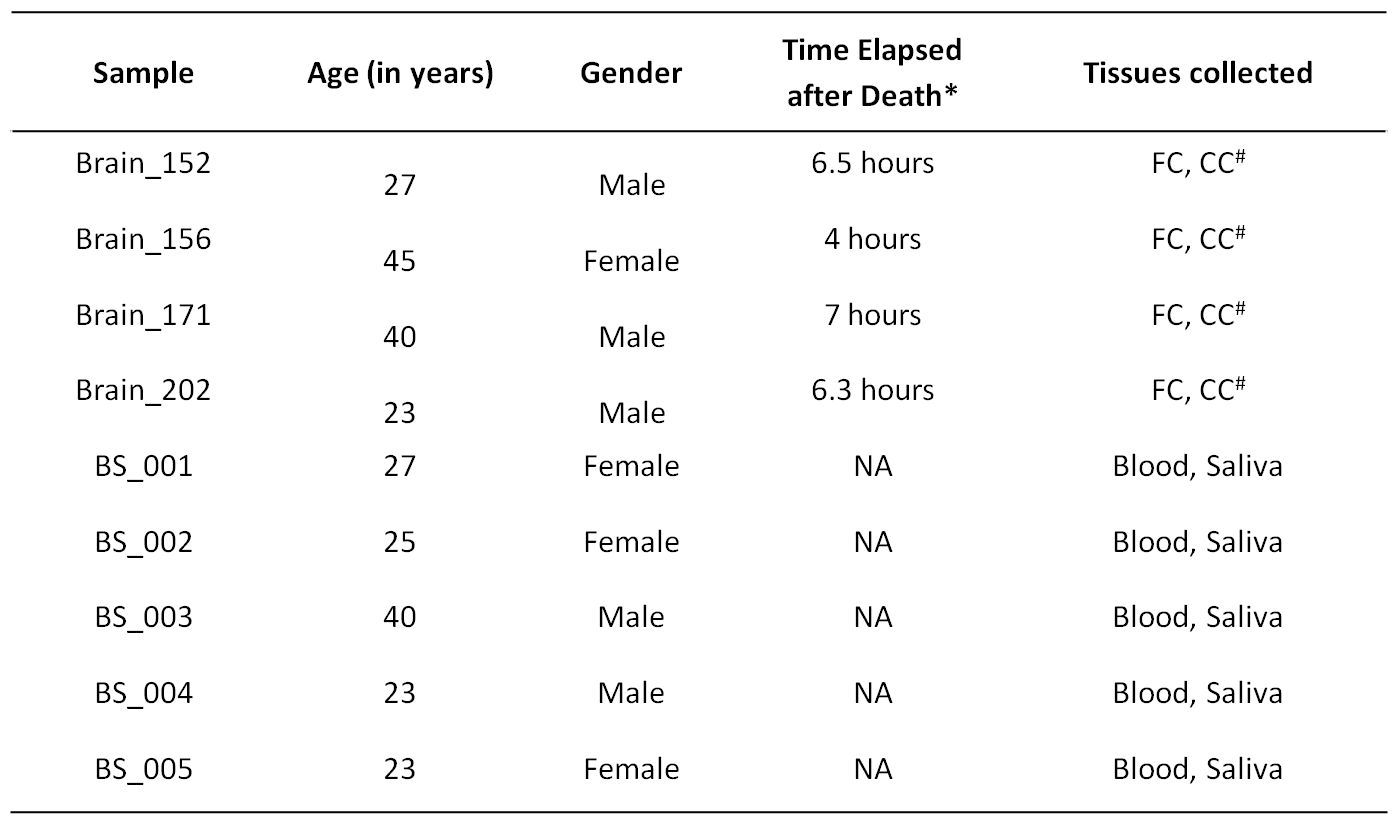
**

NA: Not Applicable, * Indicates tissue collection time, # FC- Frontal cortex, CC- Corpus Callosum (Brain Samples were collected from NIMHANS Brain Bank, Bangalore, India)

**Supplementary Table S2: Genotype concordance between somatic sites called by Varscan 2 and Mutect for brain samples**

| **Sample** | **No. of somatic sites called by Varscan 2** | **Concordance between Varscan and Mutect calls** | **Percentage concordance** |
| --- | --- | --- | --- |
| Brain_152 | 32 | 9 | 28.1 |
| Brain_156 | 132 | 104 | 78.8 |
| Brain_171 | 90 | 59 | 65.6 |
| Brain_202 | 117 | 81 | 69.2 |

**Supplementary Table S3: Genotype concordance between Exome sequence and Infinium genotyping**


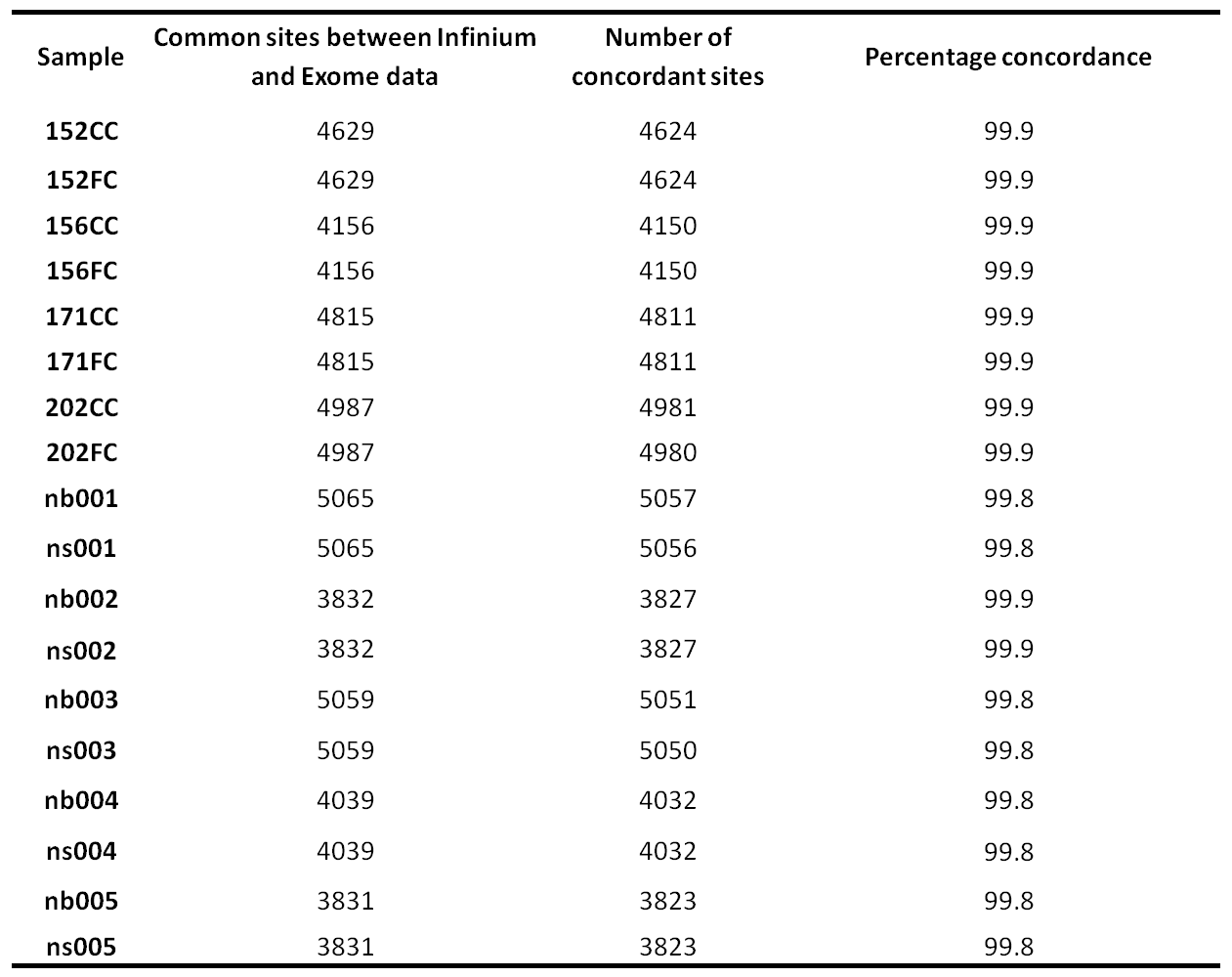


**Supplementary Table S4: Comparison of Hi-Seq and Mi-Seq data for the selected loci**


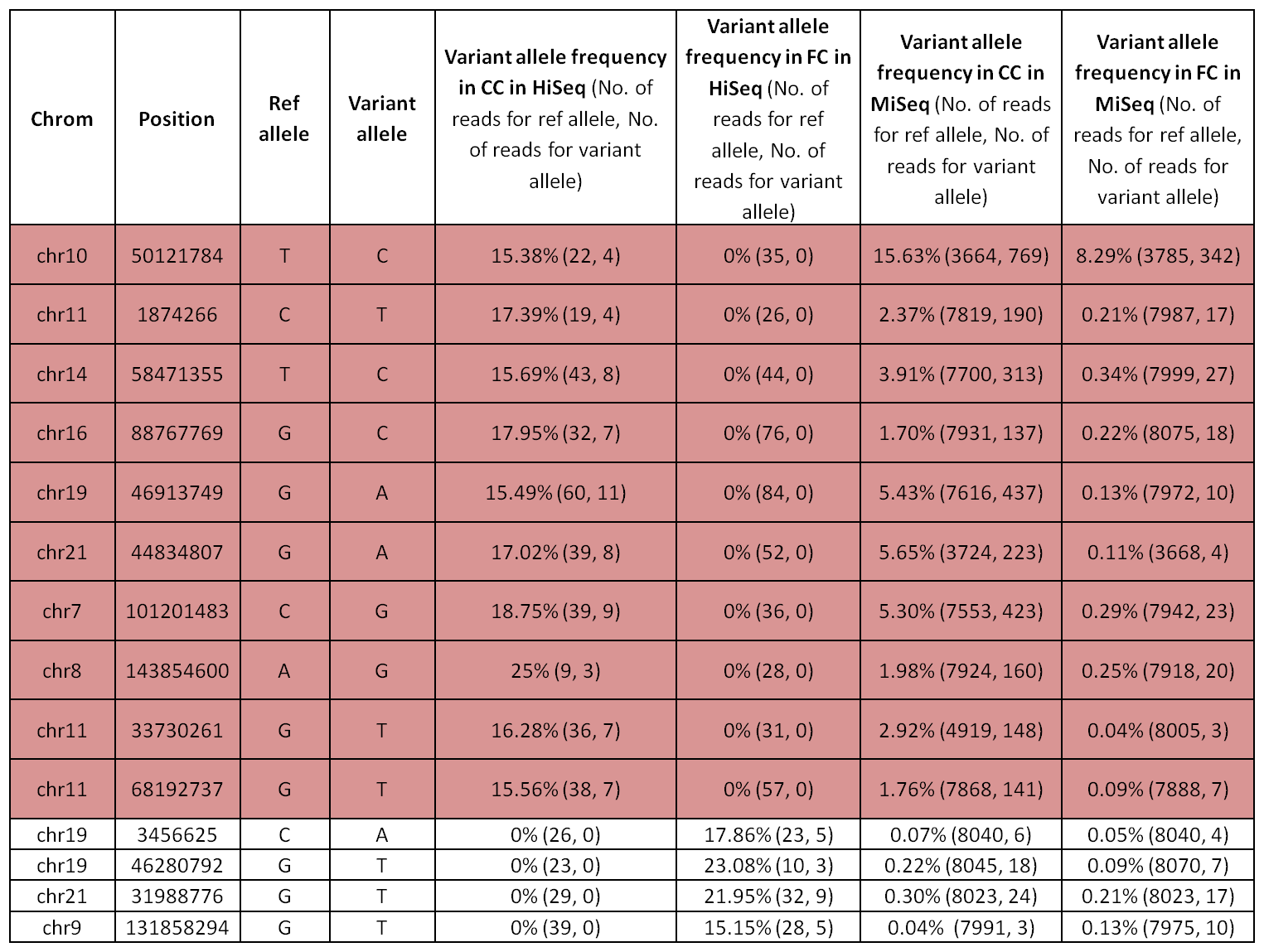


The rows marked in red indicate the validated loci

**
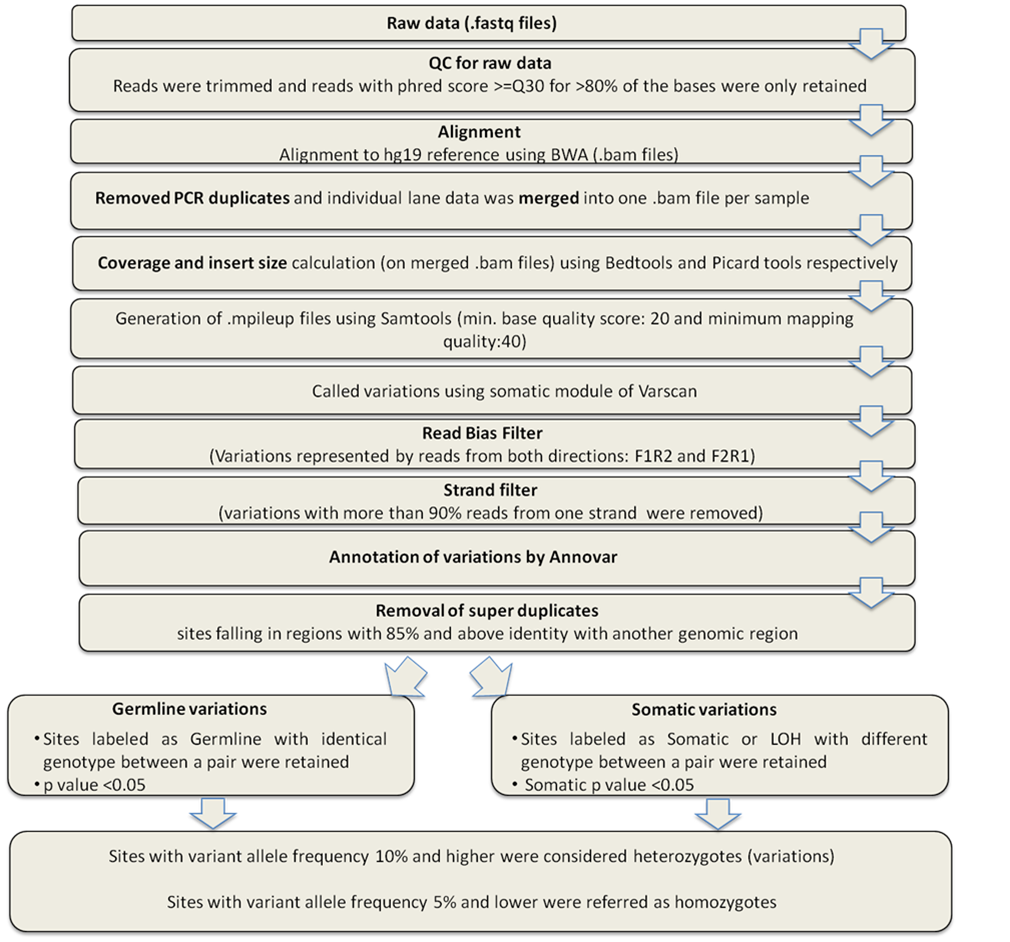
**

**Supplementary Figure S1:** Overall pipeline followed for data analysis


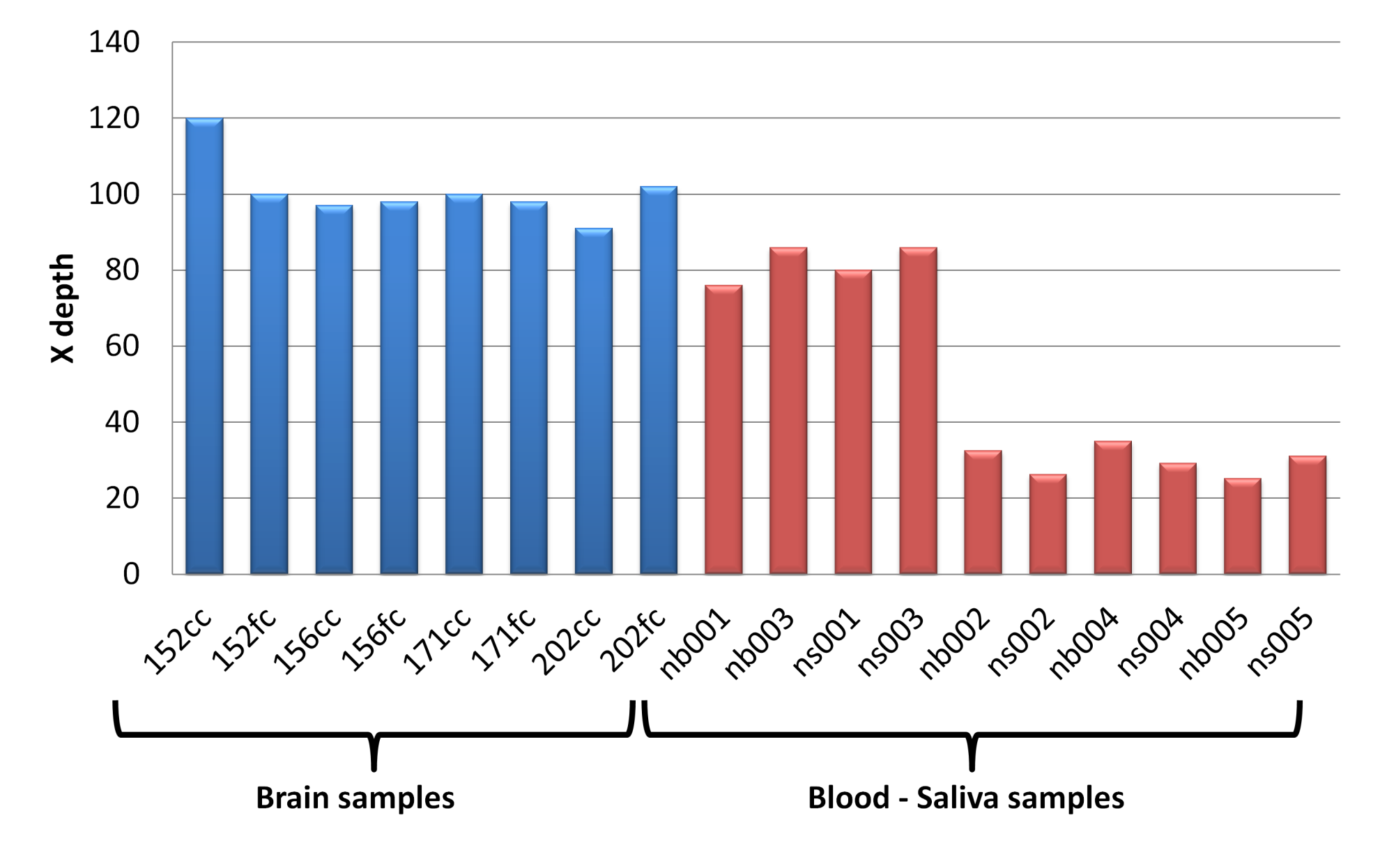


**Supplementary Figure S2:** Average read depth of all the samples: Blue bars represent average read depth for all the brain samples and red bars represent the same for blood-saliva samples.


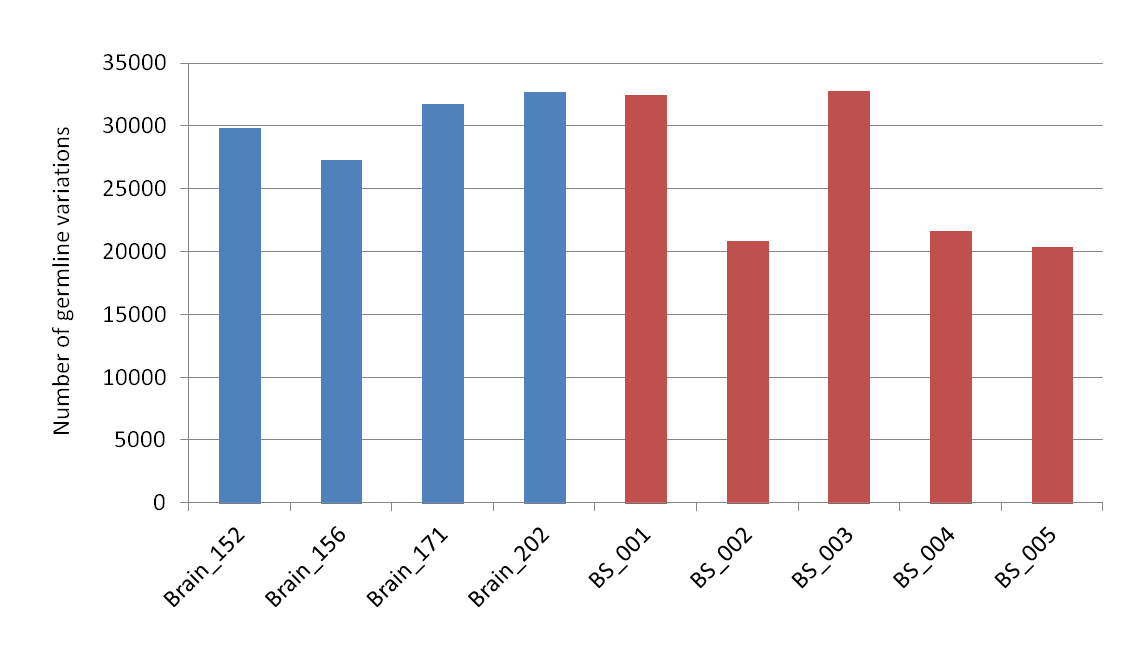


**Supplementary Figure S3: Number of germline variations per sample pair:** Variations with same genotype in both tissues from same sample are termed as germline variations, hence one bar for each sample pair. Blue bars represent brain samples and red bars represent blood saliva samples


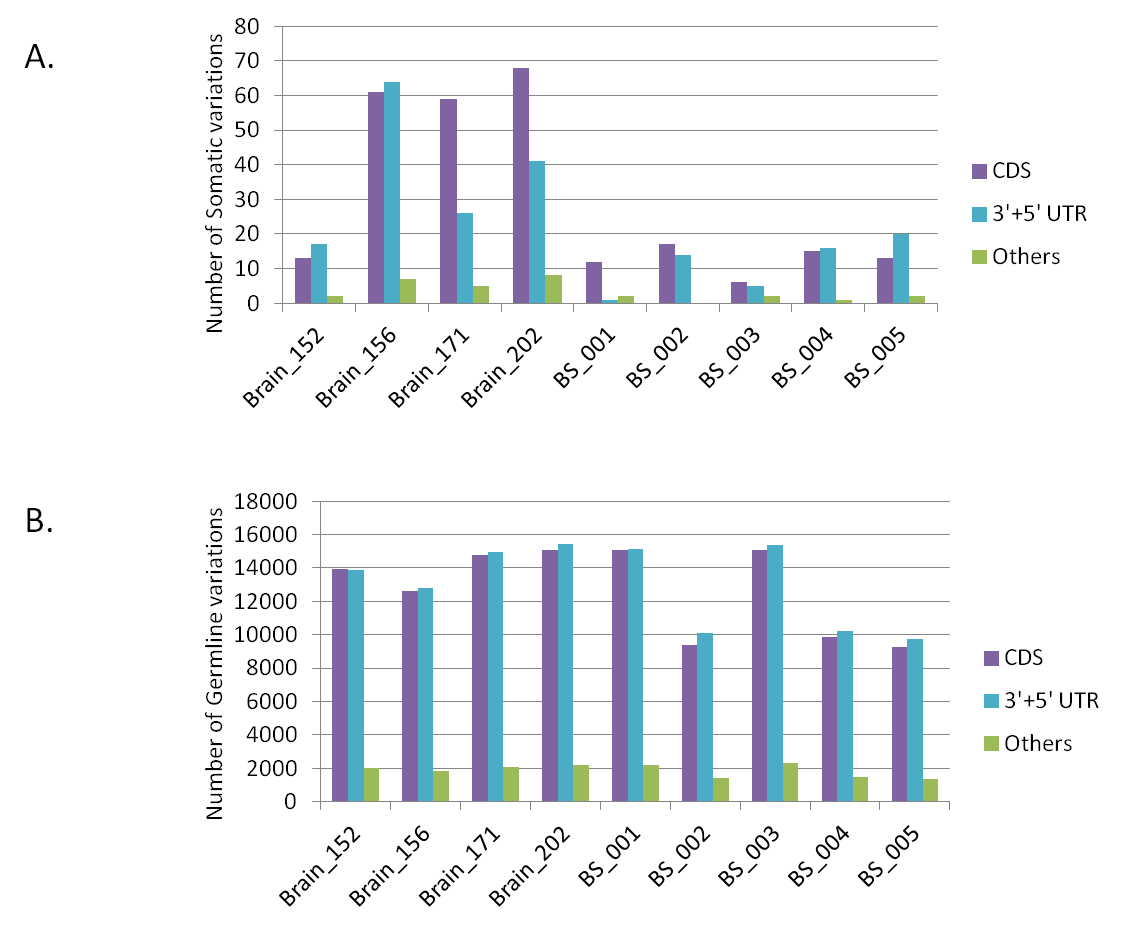


**Supplementary Figure S4:** Distribution of somatic variations (A) and germline variations (B) across different genomic regions (others: Downstream, upstream, intergenic, splicing, exonic, intronic, UTR’s of non- coding RNAs)


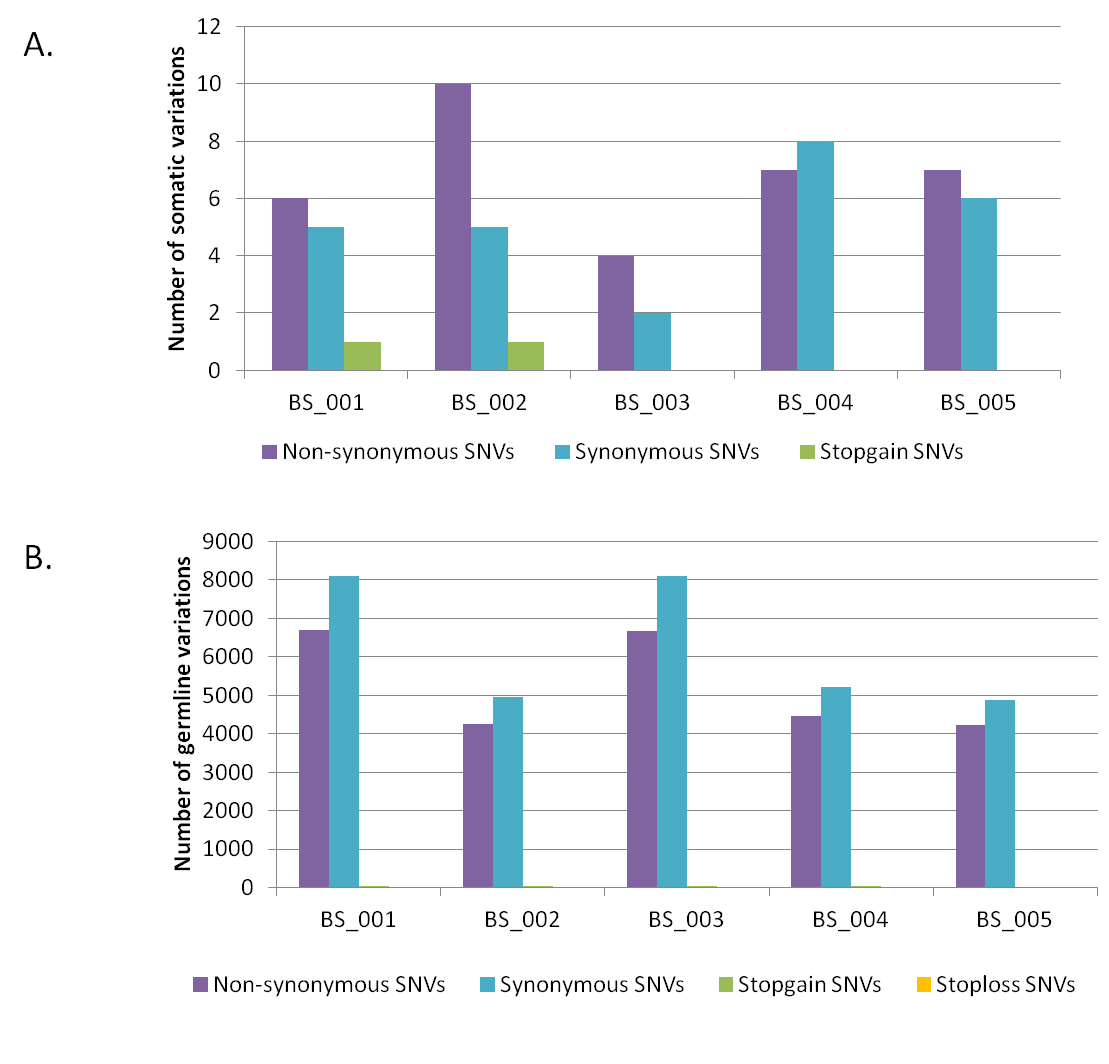


**Supplementary Figure S5**: Distribution of synonymous and non-synonymous variations in all blood-saliva samples A) for somatic variations and B) for germline variations


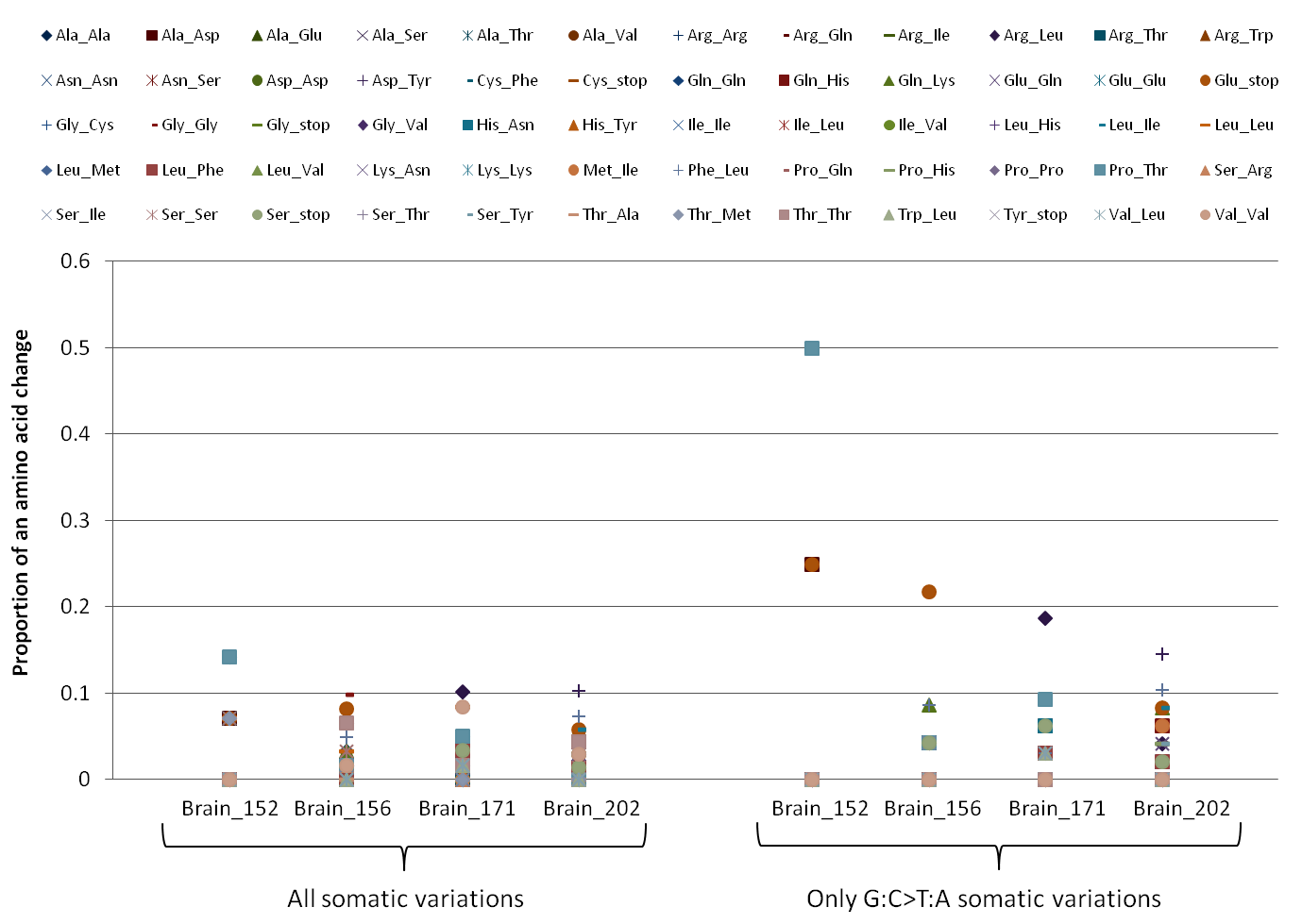


**Supplementary Figure S6**: Distribution of amino acid changes in total somatic variations and only G:C>T:A somatic variations.


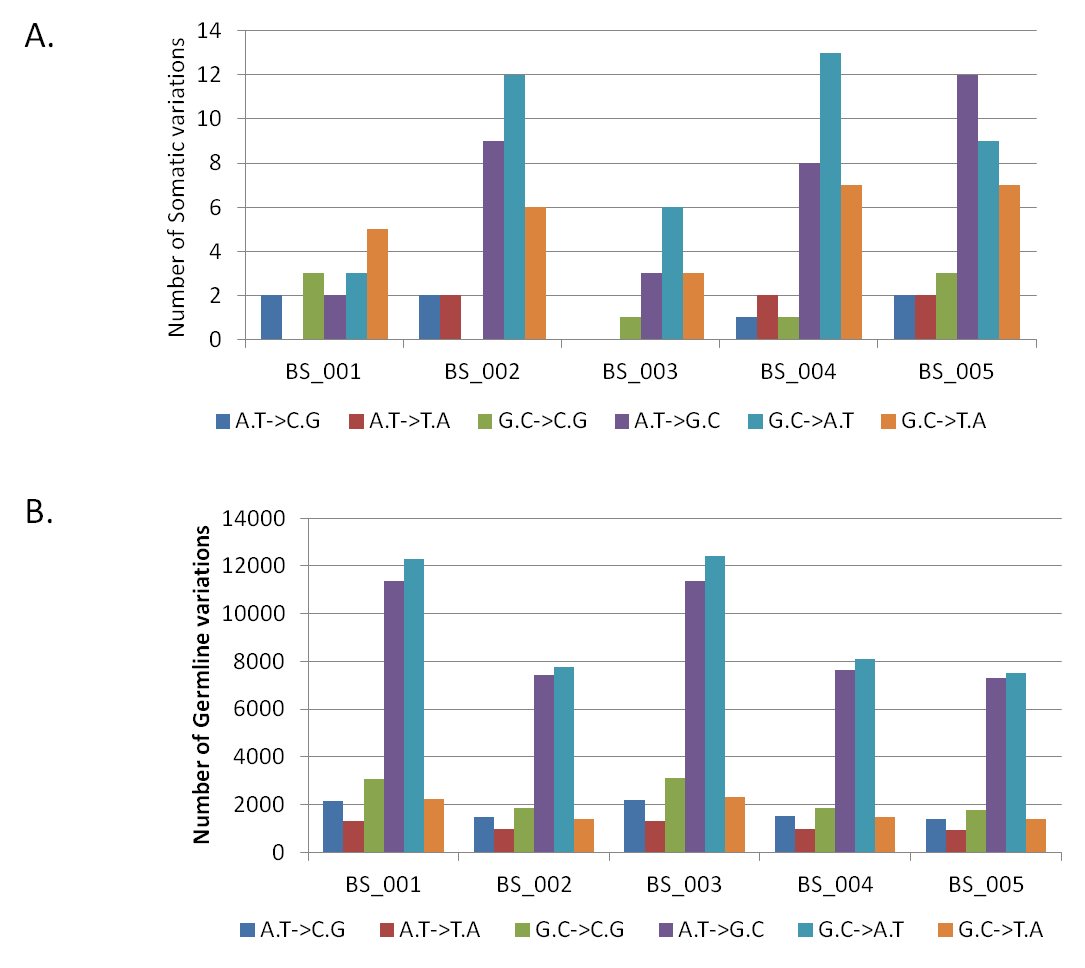


**Supplementary Figure S7:** Base change distribution in somatic (A) and germline variations (B) of blood-saliva samples


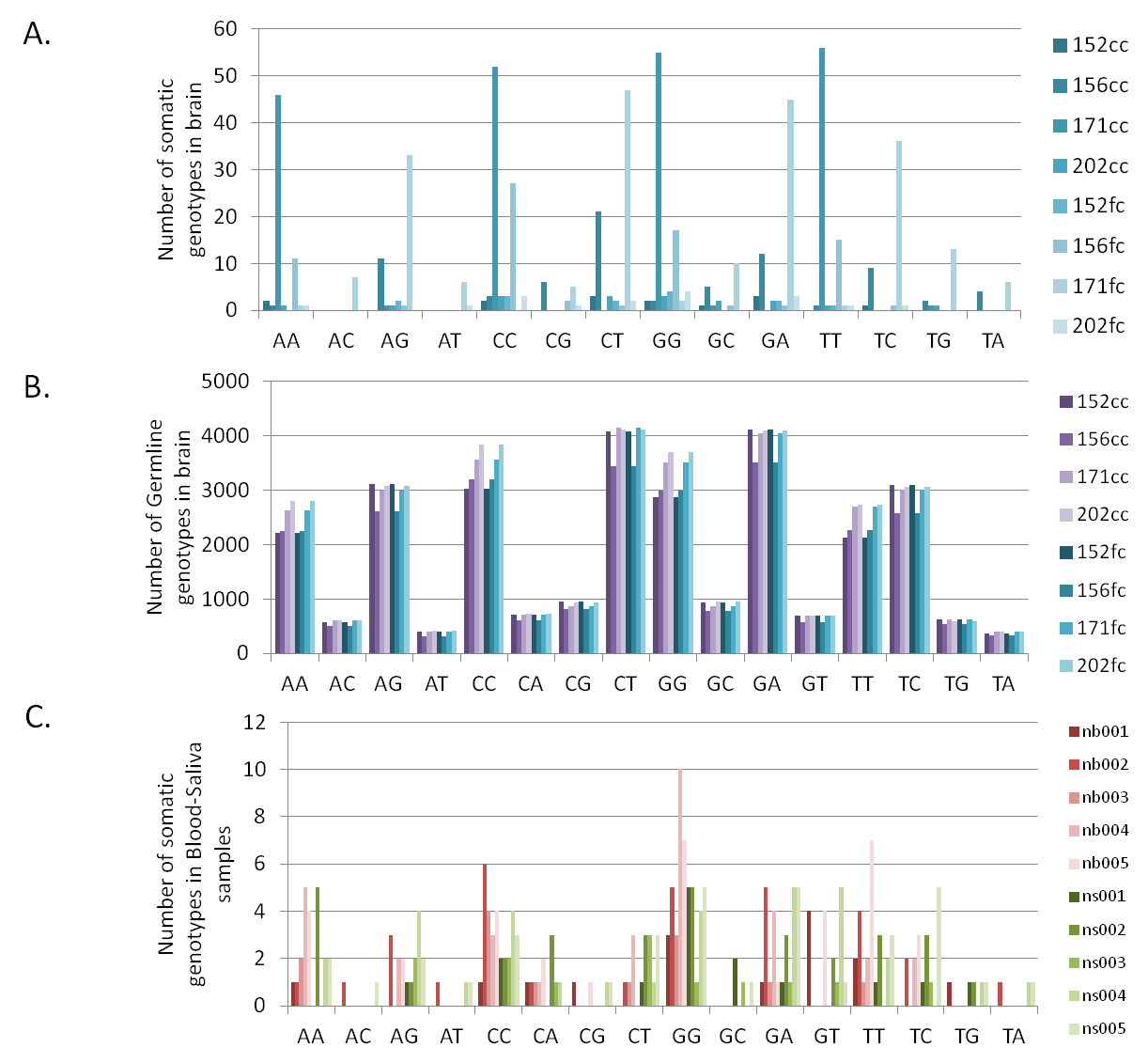


**Supplementary Figure S8:** Distribution of genotypes for variations A) for somatic variations (excluding G:C>T:A changes) in brain B) for all classes of germline variations in brain samples C) somatic variation in blood-saliva samples


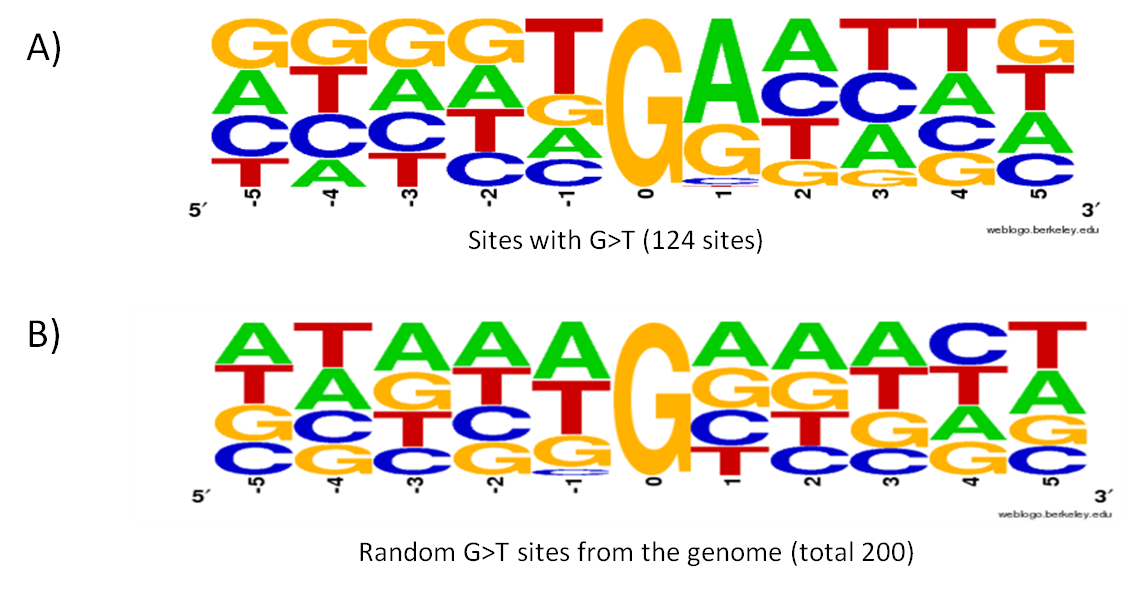


**Supplementary Figure S9: The neighbouring sequence context for G>T transversions**: A) A total of 124 sequences of 11 nucleotides each were used to generate the frequency plot. For each sequence the ‘G’ in the middle was found to have a somatic transversion (G>T) in our data. The motif shows clear enrichment of ‘A’ immediately 3’ to the ‘G’. B) From the reference genome sequence (Chromosome 1, hg19) 11 contiguous nucleotides were randomly selected with a ‘G’ in the middle (6^th^) position. A total of 1000 sets were made keeping 200 sequences of 11 nucleotides in each set and frequency plots were generated for each of these set of 200. The figure shows a representative plot. Unlike the upper panel here the bias for 3’ A is not observed.
