# Supplementary material for: Human brain harbors single nucleotide somatic variations in functionally relevant genes possibly mediated by oxidative stress
Source: F1000Res. 2017 Jan 12;5:2520. Originally published 2016 Oct 14. [Version 3] doi: 10.12688/f1000research.9495.3 (PMC5265704; doi:10.12688/f1000research.9495.3)
Supplement: Peer review history

# Peer review: Referee response for version 3

**DOI:** 10.5256/f1000research.11473.r18397

**Published:** 2017-01-20

**Authors:** Arindam Maitra

**Affiliations:** 1 National Institute of Biomedical Genomics, Kalyani, India

I see merit in the author's argument on his overall contention. I approve this with a word of caution that many of these "somatic mutations" might not be true variants but artifacts, especially oxo-G artifacts.

I have read this submission. I believe that I have an appropriate level of expertise to confirm that it is of an acceptable scientific standard.

# Peer review: Referee response for version 3

**DOI:** 10.5256/f1000research.11473.r19359

**Published:** 2017-01-16

**Authors:** Kunihiko Sakumi

**Affiliations:** 1 Division of Neurofunctional Genomics, Kyushu University, Fukuoka, Japan

The revised version of manuscript has been modified appropriately.

I have read this submission. I believe that I have an appropriate level of expertise to confirm that it is of an acceptable scientific standard.

# Peer review: Referee response for version 2

**DOI:** 10.5256/f1000research.11221.r18425

**Published:** 2016-12-13

**Authors:** Kunihiko Sakumi

**Affiliations:** 1 Division of Neurofunctional Genomics, Kyushu University, Fukuoka, Japan

The revised version of manuscript has been modified almost appropriately.

 But, still I could not read the 9 somatic variation .csv files in Dataset 1, probably due to font problem. I recommend that the authors convert the csv file into pdf (or ppt) files for worldwide readers.

See attached image

I have read this submission. I believe that I have an appropriate level of expertise to confirm that it is of an acceptable scientific standard, however I have significant reservations, as outlined above.

# Response

**Published:** 2016-12-31

**Authors:** arijit mukhopadhyay

**Affiliations:** Institute of Genomics and Integrative Biology, Delhi, India

We are sorry about the technical difficulty in reading the file. There seems to be a problem of file conversion. We have now uploaded a new .csv version of the file.

# Peer review: Referee response for version 1

**DOI:** 10.5256/f1000research.10226.r17435

**Published:** 2016-11-07

**Authors:** Arindam Maitra

**Affiliations:** 1 National Institute of Biomedical Genomics, Kalyani, India

The manuscript is well written. However, I would like to point out that oxoG artefacts can also be generated during fragmentation of DNA by sonication (standard step in the method followed by authors). Given that only 71% of these mutations could be verified (the verification was not by an orthogonal method but the same sequencing technology), I am not sure if there are some residual artefacts in the final data set (even after all filteration and taking into account the fact that both tissue and blood DNA were subjected to the same method).

I have read this submission. I believe that I have an appropriate level of expertise to confirm that it is of an acceptable scientific standard, however I have significant reservations, as outlined above.

# Response

**Published:** 2016-12-05

**Authors:** arijit mukhopadhyay

**Affiliations:** Institute of Genomics and Integrative Biology, Delhi, India

Rev Comment: The manuscript is well written. However, I would like to point out that oxoG artefacts can also be generated during fragmentation of DNA by sonication (standard step in the method followed by authors). Given that only 71% of these mutations could be verified (the verification was not by an orthogonal method but the same sequencing technology), I am not sure if there are some residual artefacts in the final data set (even after all filteration and taking into account the fact that both tissue and blood DNA were subjected to the same method).

 Ans: We understand the reviewer’s concern and in general agree with him. It is possible that if we were able to validate all the sites reported in the study we might get a good proportion (at least 30% by the validation rate) of the sites to be false positives for oxoG artefacts as well as other less known reasons. It is also true that given our stringent analysis thresholds we would also have some false negatives. Recently published estimates of somatic variations in single neurons in the whole genomes also points to this possibility. Given the scope of the present study we will not be able to accurately quantify either type of errors. However, we would like to point out that the main point of our study is not the exact locations of the somatic variations but the fact that physiological oxidative stress, in moderation, can generate such variants which have a biological significance (in terms of pathway enrichment). We hope that the reviewer will see merit in the argument.

 The reviewer is right to point out that our validation platform was not an orthogonal technique. We chose Mi-Seq over other methods considering not only the orthogonality but also robustness of the platform and error rates. Comparing all parameters Mi-Seq was our method of choice as we reasoned that even with the same sequencing chemistry, we should be able to validate true positives given a very high depth of reads obtained.

# Peer review: Referee response for version 1

**DOI:** 10.5256/f1000research.10226.r17217

**Published:** 2016-10-26

**Authors:** Kunihiko Sakumi

**Affiliations:** 1 Division of Neurofunctional Genomics, Kyushu University, Fukuoka, Japan

In the manuscript, Sharma et al. reported that adult human brains harbor single nucleotide somatic variations in the genome. Using whole exome sequencing by Illumina Truseq and Hiseq2000 system, authors compared single nucleotide variations between frontal cortex (FC) and corpus callosum (CC) of human brains. These variations were partially validated by Amplicon sequencing using MiSeq. The authors found that G to T transversion and 8-OHdG levels were increased in FC. They observed that the somatic variations were enriched in genes belonging to axon guidance pathways and neuronal processes related pathways. They propose that oxidative stress influences single nucleotide somatic variations in normal human brain.

 There is a problem with the interpretation of the data. Standard protocol of Truseq uses 100 ng of DNA, which is equivalent to 10 ^7^ human cells. To get 10% of alt sequence, 2 x 10 ^6^ cells should carry the same variation in hetero. In the adult brain tissue, it is hardly to occur the 8-OHdG caused mutation at the same site of 2 x 10 ^6^ cell genome. If this observation is correct, at least 21 times DNA replication events are required to expand the cell population carrying the same somatic variation. Such mosaicism is well known in development as described by the authors in Introduction. Probably, the seed of the observed single nucleotide variation caused by 8-OHdG was generated around birth, rapidly neuronal cells replicating stage, and not after adulthood.

 Another possibility to explain the 10% of alt sequence is contamination of blood cells in brain tissue sample. There is no quantitative purity analysis of tissue (source of DNA). If blood or other type of cell were contaminated in the FC sample, somatic mosaicism might be detectable in this system. If the author could provide the SNV of blood DNA from the same brain donor individual, we can get the answer.  

 I recommend the authors to reconsider the data, and to prepare the model including time scale of mutation accumulation reflecting these results. 

 Comments:

- Figure 4 -
  In this figure, proportion of genotype exhibits completely symmetrical pattern between CC and FC. I cannot understand the reason. The authors should discuss this observation with any possible mechanism.  

- p9, 1 ^st^ paragraph -
  To avoid inappropriate bias, authors should use high fidelity DNA polymerase for PCR, and Sanger sequencing method to validate the variant ratio, quantitatively. By showing the chromatogram pattern, we can discuss the variant ratio quantitatively.

- Dataset 1 -
  I could not read the 9 somatic variation.csv files in Dataset 1.

I have read this submission. I believe that I have an appropriate level of expertise to confirm that it is of an acceptable scientific standard, however I have significant reservations, as outlined above.

# Response

**Published:** 2016-12-05

**Authors:** arijit mukhopadhyay

**Affiliations:** Institute of Genomics and Integrative Biology, Delhi, India

Rev Comment: In the manuscript, Sharma et al. reported that adult human brains harbor single nucleotide somatic variations in the genome. Using whole exome sequencing by Illumina Truseq and Hiseq2000 system, authors compared single nucleotide variations between frontal cortex (FC) and corpus callosum (CC) of human brains. These variations were partially validated by Amplicon sequencing using MiSeq. The authors found that G to T transversion and 8-OHdG levels were increased in FC. They observed that the somatic variations were enriched in genes belonging to axon guidance pathways and neuronal processes related pathways. They propose that oxidative stress influences single nucleotide somatic variations in normal human brain.

 There is a problem with the interpretation of the data. Standard protocol of Truseq uses 100 ng of DNA, which is equivalent to 10 ^7^ human cells. To get 10% of alt sequence, 2 x 10 ^6^ cells should carry the same variation in hetero. In the adult brain tissue, it is hardly to occur the 8-OHdG caused mutation at the same site of 2 x 10 ^6^ cell genome. If this observation is correct, at least 21 times DNA replication events are required to expand the cell population carrying the same somatic variation. Such mosaicism is well known in development as described by the authors in Introduction. Probably, the seed of the observed single nucleotide variation caused by 8-OHdG was generated around birth, rapidly neuronal cells replicating stage, and not after adulthood.

 Ans: We agree with the reviewer. As it is known in the literature, for human brain the neurogenesis process starts at E13 and finishes by E108. The cell division is asymmetric, as after a certain point from each division results one mitotic and one post-mitotic neuron – and hence accurate prediction of the number of dividing neurons within this time-frame is not possible. According to the estimates an infant brain has neurons in the order of 10 ^10^ – 10 ^11^. As pointed by the reviewer, following our criteria at least 10 ^6^ cells needs to have the genomic variation. So, we also hypothesize that the variations we are observing had occurred before birth or within the first couple of years after birth. Looking at the specific type of majority of the changes (G>T transversions), it is even more compelling that this is most likely as the high metabolic demand of the cortical region would create a local oxidative stress and such ‘errors’ might have a rapid clonal expansion if these would enhance the ‘fitness’ of the local population.

 Rev Comment: Another possibility to explain the 10% of alt sequence is contamination of blood cells in brain tissue sample. There is no quantitative purity analysis of tissue (source of DNA). If blood or other type of cell were contaminated in the FC sample, somatic mosaicism might be detectable in this system. If the author could provide the SNV of blood DNA from the same brain donor individual, we can get the answer.  

 Ans: We understand the reviewer’s concern. However, we would like to argue that the observed results cannot come from contamination for the following reasons:

1. We see a specific pattern in the frontal cortex of heterozygous sites which is very different from that of corpus callosum obtained from the same brain. Such a pattern would not appear  for each sample if it was due to contamination
2. We see enrichment of relevant biological processes for each individual only for the relevant tissue type. Such a pattern is also not expected for a random contamination event

We agree that data on blood SNV for the same samples would be useful in addressing it in a more robust way – but unfortunately, such samples were not available.

 Rev Comment: I recommend the authors to reconsider the data, and to prepare the model including time scale of mutation accumulation reflecting these results. 

 Ans: We attempted to devise a model but finally could not do it as the neuronal division is asymmetric. It starts at E13 initially in a symmetric manner (i.e. two mitotic daughter cells from one mother cell) but soon becomes asymmetric (one mitotic and one post-mitotic daughter cells) which relies on a milieu of chemical cues – both inducing as well as inhibitory. Thus any model would be highly inaccurate. Instead, in the revised version we have now added the following sentences under the discussion section. We hope the reviewer will find it acceptable.

 “From our observed results, it seems that most of the variations appeared around the time of birth when neurons are rapidly dividing. It is known in the literature that the process of neurogenesis spans from E13 to E108 and the number of neurons in an infant brain is in the order of 10 ^10^ – 10 ^11^. With our analysis threshold of at least 10% abundance of the variant allele, the variation should be present in 10 ^6^-10 ^7^ cells – which is in order with the developmental time-frame. However, studies with more tissues (from brain and outside brain) from the same individuals would be needed to rule out other possible reasons.”

 Rev Comments:

- Figure 4 -
  In this figure, proportion of genotype exhibits completely symmetrical pattern between CC and FC. I cannot understand the reason. The authors should discuss this observation with any possible mechanism.  

Ans: The reason is our analysis pipeline. As described in the manuscript any variation qualifies as a somatic variation where genotype of one tissue (e.g. frontal cortex) varies from that of the other pair (e.g. corpus callosum). Hence, by definition if at a locus FC genotype is heterozygous then CC would have the homozygous genotype – and vice versa. This is why we see a mirror image of genotype distribution in figure 4.

- p9, 1 ^st^ paragraph -
  To avoid inappropriate bias, authors should use high fidelity DNA polymerase for PCR, and Sanger sequencing method to validate the variant ratio, quantitatively. By showing the chromatogram pattern, we can discuss the variant ratio quantitatively.

Ans: High-fidelity DNA polymerase was used in the amplification steps. For Sanger sequencing, majority of these sites would not be picked up due to the low abundance of variant allele. Sanger sequencing is more efficient when the variant allele frequency is 20% and above. This is why we opted for a very deep sequencing approach like Mi-Seq.

- Dataset 1 -
  I could not read the 9 somatic variation.csv files in Dataset 1.

Ans: We are sorry for the technical difficulty. We have freshly uploaded the files (New Dataset 1.zip) and hope that it is readable now.
